# Visual guidance of honeybees approaching a vertical landing surface

**DOI:** 10.1242/jeb.245956

**Published:** 2023-09-01

**Authors:** Pulkit Goyal, Emily Baird, Mandyam V. Srinivasan, Florian T. Muijres

**Affiliations:** ^1^ Experimental Zoology Group, Wageningen University & Research, 6708WD Wageningen, The Netherlands; ^2^ Department of Zoology, Stockholm University, 114 18 Stockholm, Sweden; ^3^ Queensland Brain Institute, University of Queensland, St. Lucia, QLD 4072, Australia

**Keywords:** *Apis mellifera*, Insect flight, Landing strategy, Sensory-motor flight control, Optical expansion rate

## Abstract

Landing is a critical phase for flying animals, whereby many rely on visual cues to perform controlled touchdown. Foraging honeybees rely on regular landings on flowers to collect food crucial for colony survival and reproduction. Here, we explored how honeybees utilize optical expansion cues to regulate approach flight speed when landing on vertical surfaces. Three sensory-motor control models have been proposed for landings of natural flyers. Landing honeybees maintain a constant optical expansion rate set-point, resulting in a gradual decrease in approach velocity and gentile touchdown. Bumblebees exhibit a similar strategy, but they regularly switch to a new constant optical expansion rate set-point. In contrast, landing birds fly at a constant time to contact to achieve faster landings. Here, we re-examined the landing strategy of honeybees by fitting the three models to individual approach flights of honeybees landing on platforms with varying optical expansion cues. Surprisingly, the landing model identified in bumblebees proved to be the most suitable for these honeybees. This reveals that honeybees adjust their optical expansion rate in a stepwise manner. Bees flying at low optical expansion rates tend to increase their set-point stepwise, while those flying at high optical expansion rates tend to decrease it stepwise. This modular landing control system enables honeybees to land rapidly and reliably under a wide range of initial flight conditions and visual landing platform patterns. The remarkable similarity between the landing strategies of honeybees and bumblebees suggests that this may also be prevalent among other flying insects. Furthermore, these findings hold promising potential for bioinspired guidance systems in flying robots.

## Teaser

**Summary:** Honeybees use a modular vision-based control strategy to land robustly and precisely. This resembles the landing strategy of bumblebees, and might thus be commonly used by flying insects.

## INTRODUCTION

Landing is a critical phase of animal flight as it requires precise control of flight speed with reducing distance to the surface. Poor control can result in collisions that can be detrimental to the animal, especially for honeybees that perform landings very frequently (up to a thousand landings in an hour) (Ribbands, 1949). During each landing, honeybees use visual cues to regulate their speed for ensuring a safe touchdown (Baird et al., 2013; Srinivasan et al., 2000).

As a honeybee approaches a landing surface, its motion towards the surface generates an optical expansion cue (Baird et al., 2013) in which different features (vertices, edges, etc.) in the visual field appear to move radially outwards from the point that is being approached (Edwards and Ibbotson, 2007; Gibson, 1955). Such cues are generated irrespective of the surface orientation and the direction of approach (Baird et al., 2013), and can be used to measure the relative rate of expansion (Baird et al., 2013; Lee, 1976; Wagner, 1982). This relative rate of expansion (*r*) is equal to the ratio of the velocity (*V*) with which an animal is approaching a landing surface and its distance (*y*) to that surface (*r*=*V*/*y*).

Flying animals such as insects and birds have been shown to use this expansion rate to reduce their velocity while approaching a landing surface, such that it is close to zero near the landing surface (Altshuler and Srinivasan, 2018; Baird et al., 2013, 2020; Chang et al., 2016; Goyal et al., 2021; Lee et al., 1991, 1993; Shackleton et al., 2019; Tichit et al., 2020a, 2020b; Van Breugel and Dickinson, 2012; Whitehead, 2020). In other conditions, these visual expansion cues are used by flying animals to detect rapidly approaching objects, which then elicit evasive flight manoeuvres (e.g. Santer et al., 2006; Card and Dickinson, 2008; De Vries and Clandinin, 2012; Muijres et al., 2014; Cribellier et al., 2022). Here, we focused on how flying animals use visual expansion cues to perform a landing manoeuvre.

Flying animals can land rapidly and precisely on a great variety of surfaces and objects, ranging from a horizontal surface such as the ground to vertical ones such as a wall or tree branch. During landings on horizontal surfaces, the animal can use optical expansion cues to control its descent towards the surface, but it needs to use translatory optical flow cues to control its forward flight speed (Expert and Ruffier, 2015; Franceschini et al., 2007; Srinivasan et al., 2000; Whitehead et al., 2023). During landings on vertical surfaces, the approach velocity vector towards the surface is aligned with the forward flight vector, and thus solely optical expansion cues can be used to regulate the approach flight speed (Baird et al., 2013; Goyal et al., 2021; Van Breugel and Dickinson, 2012). Here, we studied how flying animals use optical expansion cues for landing, and we therefore focused specifically on vertical surface landings. That said, optical expansion cues are generated irrespective of the surface orientation and the direction of approach (Baird et al., 2013).

Previous studies on landing manoeuvres have identified different strategies that animals use for reducing the approach velocity as they draw closer to the surface. By averaging the landing kinematics of a collection of landing manoeuvres, previous work has shown that honeybees (Baird et al., 2013), bumblebees (Chang et al., 2016; Goyal et al., 2021) and possibly also fruit flies (Baird et al., 2013; Van Breugel and Dickinson, 2012) reduce their approach velocity approximately linearly with distance to the surface. This suggests that these insects use a landing control strategy in which they aim to keep the relative rate of expansion constant throughout the landing to reduce their velocity automatically with distance.

However, we previously showed that individual bumblebees tended to regularly deviate from the approach dynamics derived from averaging all flight trajectories (Goyal et al., 2021). Instead of maintaining a constant expansion rate during the entire approach, as suggested from an analysis that averages across flight trajectories, individual bumblebees reduce their approach velocity with distance in multiple steps. During each step, they maintain a constant relative rate of expansion (referred to as a set-point). Between steps, they appear to be shifting towards higher set-points. A second study on the topic showed that, in between the flight phases at constant expansion rates, the bumblebees tended to accelerate towards the landing platform, allowing them to rapidly converge to the new optical expansion rate set-point (Goyal et al., 2022a). Using control theory modelling, it was then shown that the flight phase at the optical expansion rate set-point and the preceding transient acceleration phases result from the same visual-motor control system. This modular landing strategy of decelerating at multiple distinct set-points and accelerating during the transient phases in between these set-points results in faster landings than the simple strategy of maintaining a single optical expansion set-point (Goyal et al., 2021, 2022a).

Finally, birds – including pigeons (*Columba livia*), hummingbirds (*Colibri coruscans*) and mallards (*Anas platyrhynchos*) – tend to use yet another landing strategy whereby they hold the time-to-contact rate 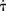 constant during their landing approach (Lee et al., 1991, 1993; Whitehead, 2020). The time to contact is defined as the instantaneous time to contact with the landing surface, should the animal continue to fly at its current approach speed, and it equals the inverse of the optical expansion rate (τ=1/*r*). How birds estimate the temporal derivative of this metric is not yet known.

Comparing the three landing strategies shows that the constant-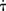 strategy of birds results in the fastest landing approach, and the single constant-*r* strategy of insects is the slowest. The stepwise constant-*r* strategy used by bumblebees has an intermediate landing duration, as it was predicted to be 16% faster than the single constant-*r* strategy and 12% slower than an equivalent constant-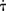 strategy (Goyal et al., 2021). What remains unclear is whether the stepwise modular landing strategy is used exclusively by bumblebees, or whether other insects such as honeybees also use it, but that it was previously unrecognized because the details of individual trajectories were not analysed. Here, we aimed to answer this question by using the individual landing analysis approach we developed previously (Goyal et al., 2021) to study the flight dynamics of honeybees landing on a vertical platform.

For this, we first used an Akaike information criterion (AIC) analysis approach to test which of the three landing strategy models fits the flight dynamics of individual honeybees best. This showed that honeybees do indeed use a modular landing strategy similar to what has been described for bumblebees. Second, we tested the dynamics of the stepwise constant-*r* landing strategy, and its robustness to variations in the environmental conditions by exploring the dependence of landing behaviour on the visual texture displayed by the landing platform. We found that the modular landing strategy allows honeybees to land robustly for a large range of initial flight conditions and visual landing platform patterns.

The striking similarities in landing strategy of honeybees and bumblebees suggest that similar strategies may also be used by other flying insects. This robust landing control system of honeybees can be used as bioinspiration for the development of landing controllers in flying robots.

## MATERIALS AND METHODS

### Animals, experimental setup and procedure

**Fig. 1.**
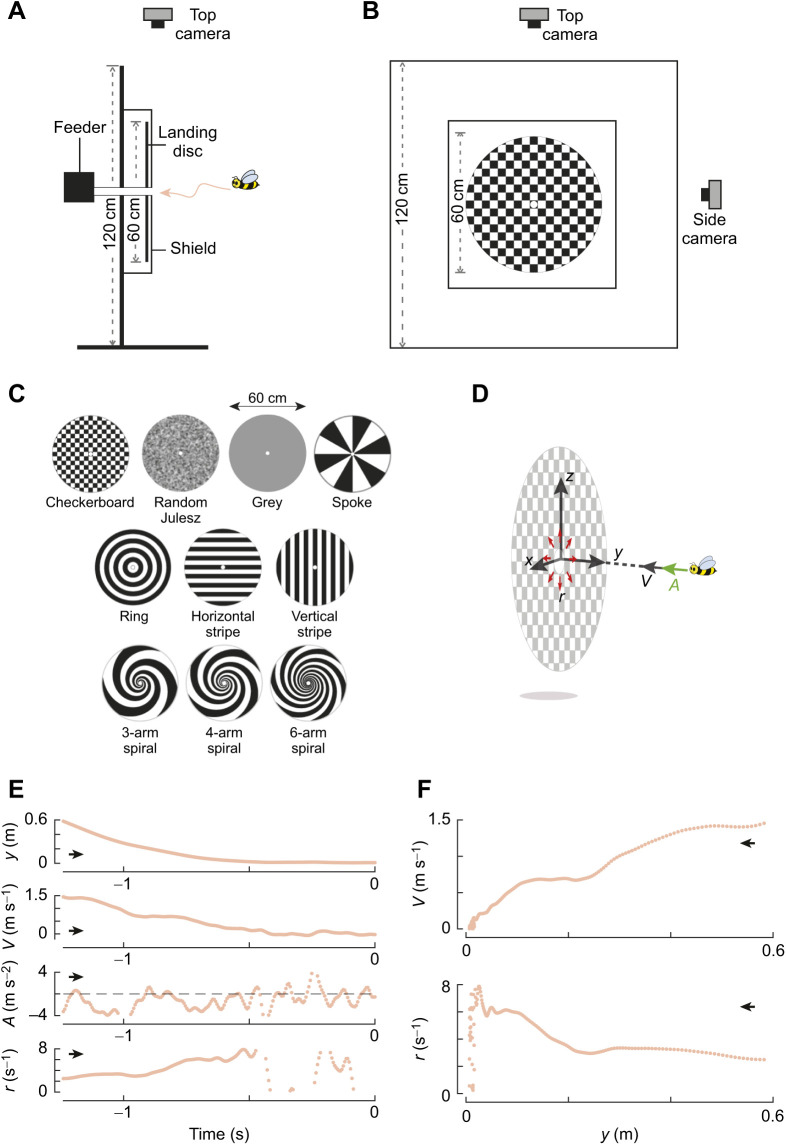
**Experimental setup, conditions and the landing kinematics of a honeybee.** (A,B) The experimental setup viewed from the side and front, respectively. The setup consisted of a flight arena with two cameras that recorded the flight manoeuvres of honeybees as they landed on a landing platform. The circular disc behind the landing platform could be replaced with discs with different graphical patterns (indicated in C) (Baird et al., 2013). (D) The landing kinematics of honeybees is described in a Cartesian coordinate system with an origin at the centre of the landing platform, the *y*-axis oriented normal to the platform, and the *z*-axis pointing vertically up. For each landing manoeuvre, we calculated the approach velocity *V*=−d*y*/d*t*, approach acceleration *A*=d*V*/d*t* and relative rate of expansion *r*=*V*/*y* that the honeybee experiences for its motion along the *y*-axis. (E) The state variables (*y*, *V*, *A*, *r*) as a function of time for a typical landing manoeuvre of a honeybee on a random Julesz pattern. (F) The change in *V* and *r* with perpendicular distance to the landing platform *y* for the same example. In E and F, the black arrow shows the direction in which abscissa data vary as a honeybee approaches the landing platform.

The experiments were carried out in an indoor facility in which air currents were minimal, the ambient temperature was maintained at 24±5°C, and the light levels, measured at the centre of the flight arena (details below), were 636±297 lx (mean±s.d.). Female foragers from a honeybee colony (*Apis mellifera ligustica* Spinola 1806) were trained to fly from their hive placed in the facility wall to a vertical landing platform connected to a food source, placed on the other side of the netted flight arena (Fig. 1A,B). The netted flight arena was 1.5×1.5×1.5 m in size, and had a 15×15 cm entrance chamber. A netting door in front of the entrance chamber allowed us to ensure that only a single honeybee entered the arena each time.

The vertical landing platform consisted of a 60 cm square Perspex transparent shield plate with a 60 cm diameter disc that could present a variety of visual patterns (Fig. 1A–C). A 15 mm diameter hole in the centre of the landing platform allowed honeybees to reach a sugar water feeder behind the platform. Honeybees that learned to fly to and land on the platform were marked with paint for individual identification.

The 60 cm disc provided the bees with visual information required for landing; we used 10 different visual patterns to study how differences in optic flow information affect landing control (Fig. 1C). Each pattern was printed using black ink on white paper and then laminated with a matt transparent sheet. The different patterns were (Fig. 1C): (1) a random Julesz pattern created by overlaying 1 cm squares with a mean luminance of 50% grey with a Gaussian filter to remove sharp contrast edges; (2) a ring pattern of 3.5 cm wide concentric black and white rings; (3) a checkerboard pattern of 3.5 cm black and white squares; (4) a spoke pattern of 12 black and white evenly spaced sectors; (5–7) a 3-arm, 4-arm and 6-arm spiral pattern, respectively (for details, see Baird et al., 2013); (8,9) 3.5 cm wide black and white vertical and horizontal stripe patterns, respectively; and (10) a homogeneous 50% grey pattern. Note that the data recorded in the presence of the ring, checkerboard, spoke, 3-arm spiral, 6-arm spiral and partly for the 4-arm spiral have previously been published by analysing the average landing manoeuvres of bees approaching each pattern (Baird et al., 2013).

While most of the patterns would provide radial expansion cues as the bees approached them, the spoke pattern and the grey texture would provide relatively weaker or no expansion cues. Similarly, all black and white patterns had sharp contrast edges between the black and white that would generate strong optic flow profiles, while the Julesz pattern provided only gradual contrast changes. The vertical and horizontal stripe patterns would only provide expansion cues in the horizontal or vertical direction, respectively. The different spiral patterns would generate different temporal frequencies of contrast changes on the eye as they were approached and were therefore used to investigate whether the landing strategy of individual honeybees was regulated by temporal frequency cues.

The landing trajectories of honeybees were analysed up to a distance of 35 cm, at which point the landing platform patterns subtended 81 deg in the frontal part of the visual field. Here, all visual patterns presented to the bees, other than the grey, exceeded the spatial resolution limit of ∼2 deg (Kirschfeld, 1973), which would enable them to resolve features ∼1 cm in diameter at 35 cm.

Before each experimental day, all honeybees were allowed to freely visit the food source for at least 48 h. During this training period, the landing platform carried a checkerboard pattern. Each experimental day consisted of several sets of at least 1 h in which bees were recorded as they approached and landed on the platform displaying the experimental pattern. During the experiments, only a single honeybee was allowed into the arena at a time. Between each experimental set, the visual pattern on the landing platform was changed. This process required about 15 min, during which honeybees were prevented from entering the setup. The landing platform patterns were changed in a randomized order over at least two different days and two different time points during the day to control for the effect of external factors, such as ambient temperature, humidity and time of day. In addition to the set number, we also recorded the sequential flight number for each individual to account for its familiarity with the visual stimulus.

The flights of honeybees approaching the landing platform during each set were recorded using a stereoscopic videography system consisting of a pair of synchronized high-speed cameras (MotionPro 10k, Redlake Inc.), recording at 400 Hz (Movies 1 and 2). The position of the bee in the resulting image sequences was digitized and calibrated into 3D coordinates using the camera calibration toolbox in Matlab (Mathworks Inc.). Only trajectories that resulted in a landing and in which the honeybees maintained consistent forward flight towards the platform were included in the analysis. This represented the vast majority of all recorded flights.

### Quantifying the state variables of each landing approach flight

We expressed the recorded landing manoeuvres of honeybees in a Cartesian coordinate system with its origin at the centre of the landing platform, *y*-axis normal to the platform, and *z*-axis oriented upwards (Fig. 1D). The landing kinematics of each manoeuvre were then defined in a space–time array of the coordinate system **X**=(*x*, *y*, *z*, *t*); here, time *t* equals zero at the end of the trajectory, i.e. when the honeybee reached the landing surface. The kinematics data were post-processed using the following steps. First, to allow the use of the custom analysis tools from Goyal et al. (2021) on the data, we reduced the temporal dynamics of the landing to 175 Hz using modified Akima cubic Hermite interpolation (*makima* in Matlab 2020a). See Supplementary Materials and Methods, ‘Extraction of set-points of relative rate of expansion’ for details. Second, to reduce the tracking noise from these manoeuvres, we filtered the kinematics data using a low-pass second-order two-directional Butterworth filter (*filtfilt* in Matlab 2020a) with a cut-off frequency of 20 Hz (Fig. S1). Finally, we determined the corresponding velocity vector **U**=(*u*, *v*, *w*) and acceleration vector **A**=(*a_x_*, *a_y_*, *a_z_*) of each landing using numerical differentiation with a second-order central differencing scheme.

To determine how honeybees land, we used five state variables that describe the movement in the direction normal to the landing platform (Fig. 1D). These variables are: normal distance from the landing platform *y*(*t*), flight velocity towards the platform *V(t)=−v*(*t*), acceleration towards the platform *A*(*t*)=*−a_y_*(*t*), the relative rate of image expansion that a honeybee experiences during its landing approach *r*(*t*)=*V*(*t*)/*y*(*t*), and the equivalent time to contact τ(*t*)=1/*r*(*t*). Here, we used the velocity perpendicular to the platform for the computation of relative rate of expansion as honeybees needed to progressively reduce this component as they advanced towards the landing platform. As the velocity perpendicular to the platform was much larger than the velocities in other directions, similar results are obtained when the three-dimensional velocity is used to compute the relative rate of expansion.

**Fig. 2.**
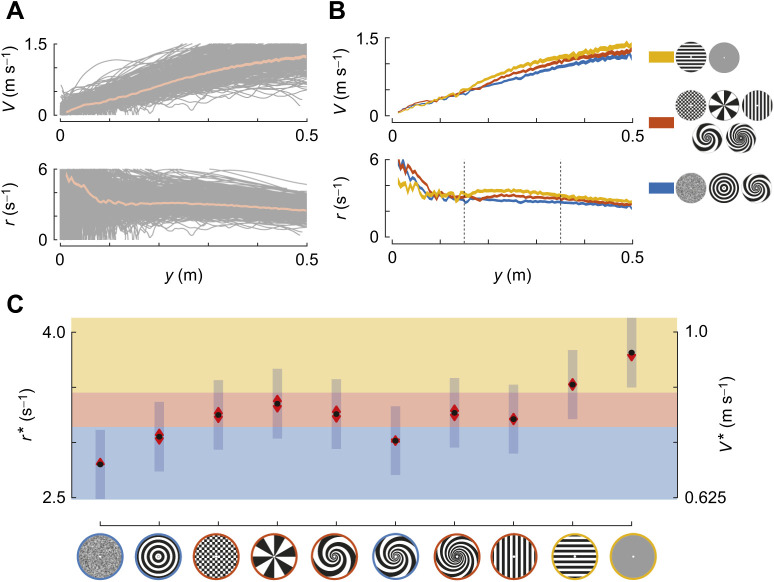
**On average, honeybees decrease their approach velocity linearly with the perpendicular distance to the landing platform.** (A,B) Approach velocity *V* and relative rate of expansion *r* with perpendicular distance to the landing platform *y* for (A) all recorded landing manoeuvres and (B) the approaches averaged over three different landing platform pattern clusters, as defined on the right of B. The orange curve in A and all curves in B show the average approach dynamics, whereby the thickness of these curves represents the standard error of the mean. All landing tracks are shown in grey in A. The three landing platform pattern clusters (right of B) were defined based on the average set-point of relative rate of expansion r* that the patterns elicit in the bees (C). The low-r* cluster (blue) consists of the random Julesz, rings and 4-arm spiral patterns. The high-r* cluster (yellow) consists of the horizontal stripes and grey pattern. All other patterns fall within the intermediate-r* cluster (orange). See Materials and Methods for details on the cluster definition and selection procedure. (C) The average set-point of relative rate of expansion r* for the different landing patterns as predicted by a linear mixed-effects model (Table 1). The right axis shows the corresponding average approach velocity V* at distance *y*=0.25 m from the landing platform. Black dots depict estimated means, vertical blue bars are 95% confidence intervals and red arrows head show whether r* and V* differ significantly among landing patterns (no overlap indicates statistically significant differences). The average set-points of relative rate of expansion r* for each landing pattern were determined within the distance range of 0.15 m≤*y*≤0.35 m, indicated in B. The number of landings and sampled individuals per treatment are given in Table S1.

To determine how landing honeybees regulate their approach speed based on optical expansion information, we used an analysis method previously developed for analysing the landing dynamics of bumblebees (Goyal et al., 2021). This analysis method was based on a functional model of the visual-motor insect flight control system, whereby flying at a constant optical expansion rate would result in a linear reduction in flight speed with reducing distance from the landing platform (Baird et al., 2013; Goyal et al., 2021). Thus, flying at a constant optical expansion rate would consequently result in a smooth landing (Fig. 2A,B).

**Table 1.**
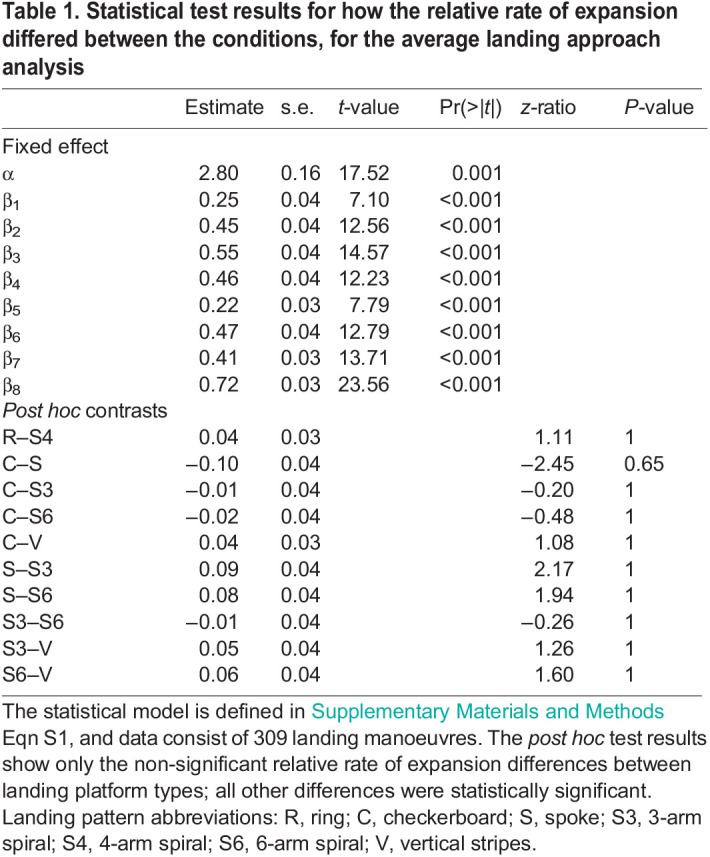
Statistical test results for how the relative rate of expansion differed between the conditions, for the average landing approach analysis

**Fig. 3.**
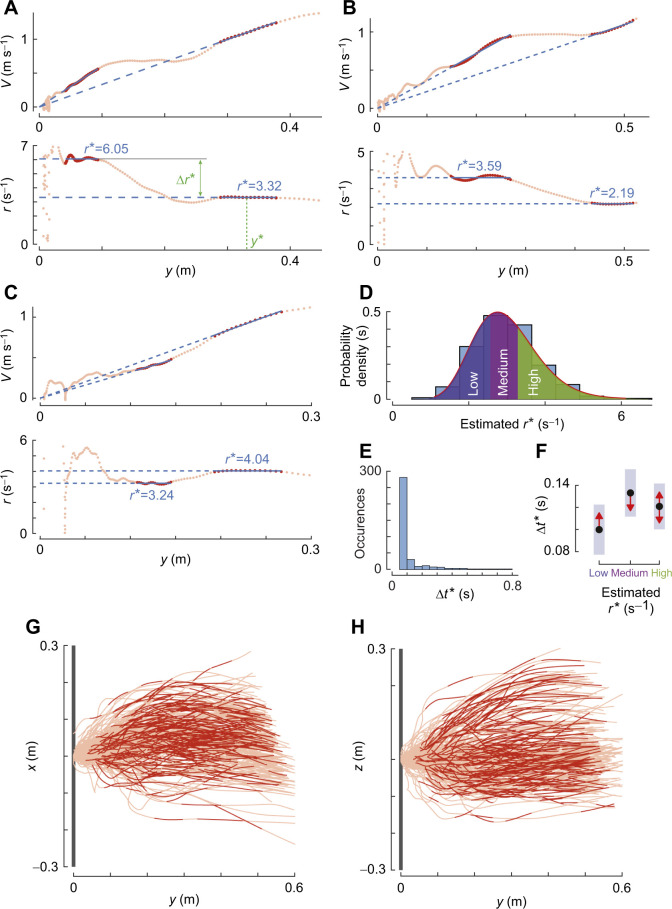
**Honeybees exhibit a range of set-points during a single landing manoeuvre.** (A–C) Approach velocity *V* and relative rate of expansion *r* with perpendicular distance to the landing platform *y* for three different landing manoeuvres, including (A) the example shown in Fig. 1E,F. We used an automatic detection algorithm (Goyal et al., 2021) to identify the track segments in which honeybees held *r* approximately constant. The identified constant-*r* segments are highlighted in red, and corresponding values of the expansion rate set-point r* are shown in blue (as slopes and ordinate values in the top and bottom graph of each panel, respectively). (A) For the first constant-*r* segment, the mean distance to the landing platform is defined as y*; the change in the set-point between the two consecutive constant-*r* segments is defined as Δr*. (D) Probability density of all identified set-points (*n*=359) in all recorded landing manoeuvres. The observed probability density is approximated using a gamma distribution (red curve) and divided into three groups: low expansion rate set-points (r*≤2.58 s^−1^), medium set-points (2.58<r*≤3.29 s^−1^) and high set-points (r*>3.29 s^−1^). Each group has an equal one-third probability of occurrence. (E) Histogram of time travelled during a constant-*r* segment Δt* for all 359 constant-*r* segments. (F) Flight duration at a set-point Δt* in low, medium and high r* set-point groups defined in D (Table S2). Black dots depict estimated means, vertical blue bars are 95% confidence intervals and red arrows show whether Δ*t* differs significantly between groups (no overlap indicates statistically significant differences). (G,H) Top and side view, respectively, of the trajectories of all 309 recorded landing manoeuvres, showing the lateral position *x* and the vertical position *z*; the sections identified as constant-*r* segments are highlighted in red.

As part of this analysis method, we therefore used an algorithm that identifies the landing approach segments in which honeybees held the relative rate of optical expansion (*r*) constant as they decelerated toward the landing platform. These track segments and constant values are referred to as constant-*r* segments and the corresponding set-points of relative rate of expansion (r*), respectively. The set-point value (r*) was estimated by computing the mean relative expansion rate in a constant-*r* segment (Fig. 3A–F). Additionally, we computed for each constant-*r* segment the mean approach velocity (V*), the mean distance of the honeybee from the landing platform (y*), the distance travelled during the constant-*r* segment (Δy*), and the time duration of the constant-*r* segment (Δt*). Thus, the dynamics of each constant-*r* segment was identified using the parameter set (r*, V*, y*, Δy*, Δt*). For landing approaches that contained two or more constant-*r* segments, we also identified the step-change in set-point that occurred between two consecutive constant-*r* segments (Δr*) (Fig. 3A).

### Comparing the three landing strategy models

**Fig. 4.**
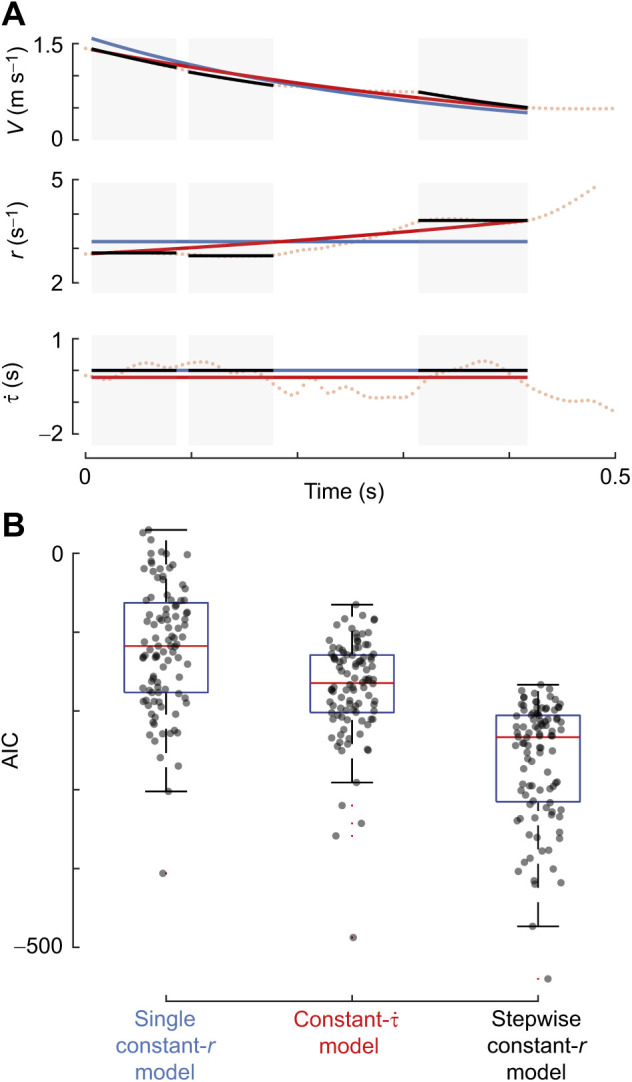
**The stepwise constant-*r* model explains the approach kinematics of landing honeybees best.** (A) The temporal dynamics of approach velocity *V*, relative rate of expansion *r* and time to touchdown 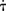 for a honeybee landing on a vertical landing platform (dotted line), with various model fits (solid lines). The fitted models are of the single constant-*r* approach model (blue), the constant-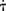 approach model (red) and the stepwise constant-*r* approach model (black lines with blue shaded background). (B) The Akaike information criterion (AIC) of the three models applied to all landing manoeuvres in which two or more stepwise constant-*r* segments were identified (*n*=100 landing approach flights). Lower AIC values show a better fit, and thus the stepwise constant-*r* model provides the best model fit.

This parameter set (r*, V*, y*, Δy*, Δt*, Δr*) allowed us to quantify the landing kinematics based on the stepwise constant-*r* model used by landing bumblebees (Goyal et al., 2021). To test how well this model captures the landing dynamics of honeybees, we compared its performance with that of the two alternative landing strategies, the single constant-*r* model for insects (Baird et al., 2013; Chang et al., 2016; Van Breugel and Dickinson, 2012) and the constant-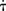 model for birds (Lee et al., 1991; 1993; Whitehead, 2020). For this, we fitted both alternative models to all flight sequences that contained two or more constant-*r* segments (Fig. 4A). For the single constant-*r* model, we determined the single optical expansion rate set-point (r*_1_), and for the constant-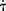 model we estimated the equivalent constant time-to-contact rate (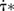). For each analyzed flight sequence, we then estimated the Akaike information criterion (AIC) per model (Bozdogan, 1987; Ljung, 1999), based on the temporal dynamics of approach flight speed. We used the AIC selection criterion to find which model captures the observed landing kinematics best. Note that in the AIC calculations, the stepwise constant-*r* model is penalized as it consists of multiple fitting parameters, which is equal to the number of identified constant-*r* segments.

### Identifying the constant-*r* segments

We identified the constant-*r* segments in a landing trajectory using an analysis method developed for landing bumblebees (Goyal et al., 2021). For this method, the identification of the constant-*r* segments depends on the factor *f*, which sets how much oscillation around the constant-*r* set-point is allowed. As a result, increasing *f* leads to more identified constant-*r* segments, but it also increases the possibility of false positives in the set. Here, we present the results for *f*=1.5, but our results remain similar for a wide range of *f* (0.5≤*f*≤2.5). For exact details about the constant-*r* detection algorithm and the independence of results with factor *f*, see Supplementary Materials and Methods, ‘Extraction of set-points of relative rate of expansion’, Fig. S2 and Table S1.

The algorithm used to find constant-*r* segments does not capture all the set-points at which honeybees fly during landing. This can be due either to the factor *f* limiting the variation in *r* for a segment to be identified as a constant-*r* segment, or to a fundamental limitation of the algorithm itself. This limitation arises because the algorithm can identify a set-point only if the honeybee flies at it for a long enough time period. In many cases, though, a honeybee may not reach the set-point; for example, because it changes the set-point before reaching its previous set-point. This problem can mostly be overcome by increasing the sample size of the study (Goyal et al., 2021). Here, we analysed hundreds of landing manoeuvres of honeybees, which should be enough for the individual-based analysis approach.

### Statistical analysis

We used R 4.0.3 (http://www.R-project.org/) for all statistical analyses. Previous analyses have shown that inter-individual variation is similar to the intra-individual variation for landing approaches of honeybees (Baird et al., 2013). Therefore, we treated each flight as an independent data point, even when we analysed multiple landings from the same bee (Table S1). In all models, we used the flight number and set number as random factors to account for learning and time of day, respectively. We used the *lmer* function in R to develop different linear mixed-effects models and to perform Bonferroni corrections to adjust the statistically significant values for comparison among means of different groups. *P*-values <0.05 were considered statistically significant.

In our statistical analysis, the maximum variance corresponding to flight number random factor in all of our individual trajectory-based analyses was less than 3×10^−5^. This signifies that learning accounted for an insignificant variation in the data, and therefore we could safely ignore any learning effects.

The landing platform patterns elicit different landing behaviour in the bees. To differentiate between these, we grouped the platform patterns into three clusters, based on the set-point of relative rate of expansion r* that the bees exhibited. The low-r* cluster (blue) is defined as the pattern cluster with r* significantly different from the highest r* pattern, and similarly the high-r* cluster is defined as the pattern group with r* significantly different from the lowest r* pattern. All other patterns fall within the intermediate-r* cluster.

See Supplementary Materials and Methods, ‘Statistical models’ for details of all statistical models.

## RESULTS

Using our experimental setup, we recorded stereoscopic videos of 309 honeybees landing on vertical platforms with variable visual patterns (see Supplementary Materials and Methods and Table S1; see also Database S1 in Dryad, https://doi.org/10.5061/dryad.ghx3ffbsj). From these videos, we reconstructed the three-dimensional flight kinematics of all landing manoeuvres. Based on these, we examined the average and individual landing approaches of honeybees, to study how honeybees land on a vertical platform with a variable visual pattern.

### Average landing approach of honeybees on a vertical landing platform

We first analysed how the average honeybee controlled its approach velocity as it advanced towards the landing surface. We did this for all recorded landing manoeuvres combined (Fig. 2A) and for the average landing per treatment (landing platform pattern) (Fig. 2B,C). For all average flights, we found that honeybees reduced their mean approach velocity *V* approximately linearly with the perpendicular distance to the landing surface *y*. This average analysis suggested that the honeybees approached the landing surface by keeping the relative rate of expansion nearly constant at a set-point r*=3.26±0.16 s^−1^ (mean±s.e.m.), as estimated within the distance range of 0.15≤*y*≤0.35 m.

To determine how the set-point r* varied among different landing patterns, we used a linear mixed-effects model on the r* data of all manoeuvres (Table 1, Fig. 2). Honeybees exhibited the highest expansion rate set-point and thus flew fastest towards the landing platform with the horizontal striped pattern (r*=3.52±0.16 s^−1^) and the grey pattern (r*=3.81±0.16 s^−1^). In contrast, they exhibited the lowest set-point and thus flew slowest towards the landing platforms with the random Julesz pattern (r*=2.80±0.16 s^−1^), rings (r*=3.05±0.16 s^−1^) and the 4-arm spiral patterns (r*=3.02±0.16 s^−1^). When landing on the platforms with the other visual patterns, the set-point r* fell between these two groups (Fig. 2B,C).

### Landing approach kinematics of individual honeybees

**Fig. 5.**
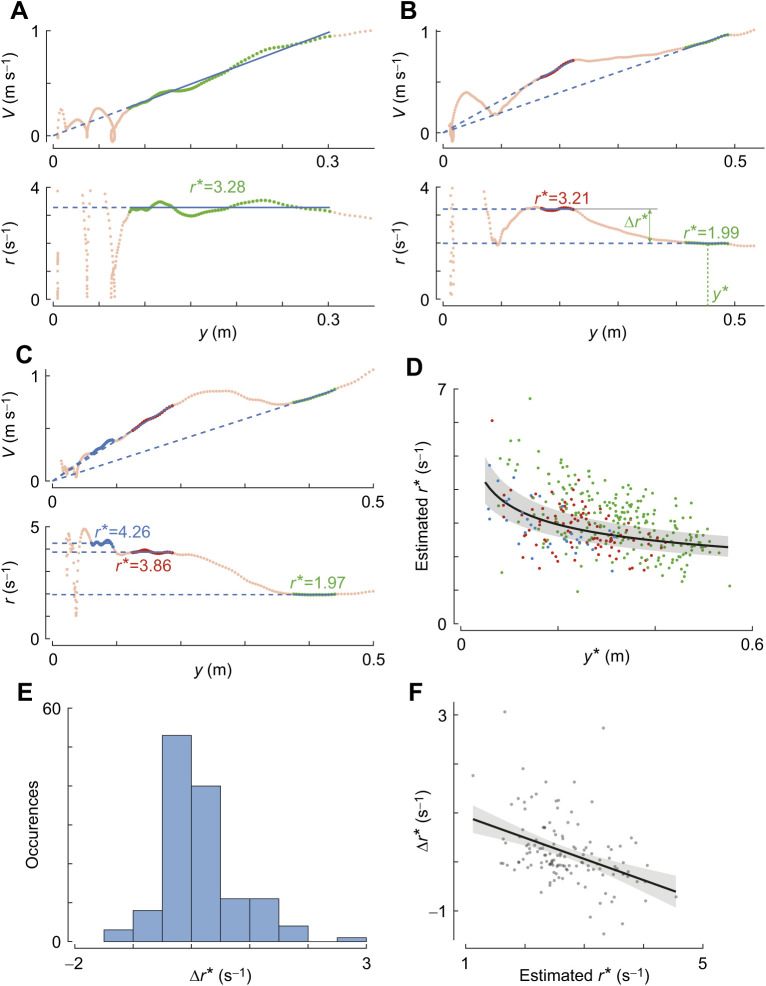
**Throughout a landing manoeuvre, honeybees regularly adjust their set-point of optical expansion rate in a stepwise manner.** (A–C) Approach velocity *V* and relative rate of expansion *r* with perpendicular distance to the landing platform *y* for various landing manoeuvres: (A) a landing in which the honeybee continued to fly at a single optical expansion rate set-point; (B) a landing in which the bee switched the approach halfway through to a higher set-point; (C) a landing in which the bee flew at three distinct optical expansion rate set-points. (D) The set-points of optical expansion rate r* with the corresponding mean distance to the landing platform y*, as defined in B. The data points show results for all identified constant-*r* segments, whereby the first, second and third (or higher) constant-*r* segments identified in a landing manoeuvre are highlighted in green, red and blue, respectively (*n*=359 constant-*r* segments). The shaded curve shows the (r*−y*) trend predicted by the linear mixed-effects model (black) with 95% confidence intervals (grey). See Table 2 for statistical results. (E) Histogram of change in the set-point Δr* between two consecutive constant-*r* segments as defined in B (*n*=132 set-point changes identified in 100 tracks with two or more set-points). (F) The change in the set-point Δr* with the set-point magnitude r*. The data points show results for all identified set-point changes, and the shaded curve shows the statistical model output (black) and 95% confidence intervals (grey). See Table S3 for statistical results.

In contrast to the continuous average landing behaviour, we observed that, for many individual landing manoeuvres, the relative rate of expansion was not constant around one set-point. Instead, honeybees often seemed to land by flying at multiple set-points (Figs 1F, 3A–C and 5B,C), although landings with a single set-point were also observed (Fig. 5A). This suggests that landing honeybees might use a stepwise constant-*r* landing strategy, similar to the one described for bumblebees (Goyal et al., 2021).

**Table 2.**
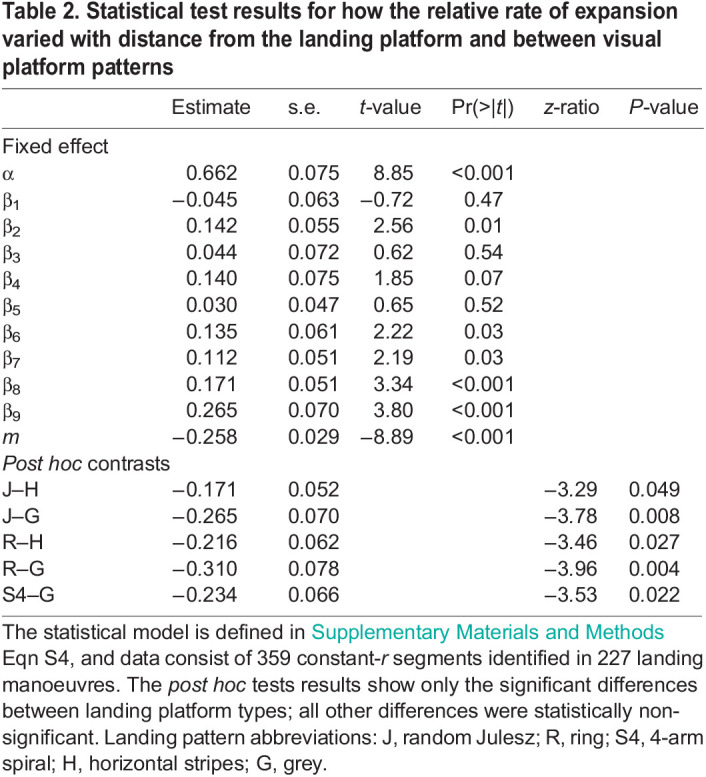
Statistical test results for how the relative rate of expansion varied with distance from the landing platform and between visual platform patterns

To test this, we compared how well the three landing strategy models described in the literature explain the landing kinematics of individual honeybees (Fig. 4). The AIC-based model selection analysis shows that the single constant-*r* landing strategy for insects performs worst, followed by the constant-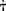 model. The stepwise constant-*r* landing strategy has the lowest AIC-values, showing that this model explains the observed landing kinematics of honeybees best (Fig. 4B). Based on these results, we selected the stepwise constant-*r* landing strategy as the most likely model for landing honeybees, and all subsequent analyses were performed using this model.

### Stepwise constant-*r* landing approach strategy of honeybees

To analyse the stepwise constant-*r* landing dynamics in detail, we extracted all track segments in which honeybees kept the relative rate of expansion nearly constant using the detection algorithm we developed previously (Goyal et al., 2021) (Fig. 3), and determined the parameter set (r*, V*, y*, Δy*, Δt*) for each segment. By analysing these data, the following results were obtained.

#### Honeybees exhibit a range of set-points and travel longer near the medium set-points as compared with the low set-points

For the detection threshold factor *f*=1.5 in the set-point extraction algorithm, we identified 359 constant-*r* segments within 227 of the 309 recorded landing tracks (Fig. 3G,H). For the sensitivity for our results to a wide range of detection threshold factor *f*, see Fig. S2 and Table S1. The identified set-points of relative rate of expansion r* in these constant-*r* segments varied within a wide range (Fig. 3D). The observed distribution of r* can be approximated by a gamma distribution (median r*=2.93 s^−1^, *a*=­­12.80 [11.14–14.87], *b*=0.23 [0.20–0.27], mean [95% confidence intervals]) (Evans et al., 2000).

To identify whether honeybees possessed an inclination to fly at certain set-points, we divided the observed distribution of set-points into three groups with equal probability: low-r* group (r*≤2.58 s^−1^), medium-r* group (2.58<r*≤3.29 s^−1^) and high-r* group (r*>3.29 s^−1^). We then used a linear mixed model to test whether the duration for which the bees flew near their set-point (Δt*) differed between these three set-point groups (Fig. 3E,F; Table S2). We found that honeybees in the medium set-point group exhibited a 33% longer travel time than those in the lowest r* set-point group (low-r* group: Δt*=0.10 s; medium-r* group: Δt*=0.13 s; Table S2, *P*=0.037). The travel time in the highest set-point group did not differ significantly from others, and the landing patterns also did not significantly affect travel time (Table S2).

We then tested whether this variation in Δt* also caused a variation in distance travelled (Δy*) between the three set-point groups (Table S2). This linear mixed model shows that honeybees in the medium and high set-point groups travelled 31% and 33% longer distances during their constant-*r* segment compared with the low set-point group (low-r* group: Δ*y*=0.069±0.006 m; medium-r* group: Δ*y*=0.099±0.006 m; high-r* group: Δ*y*=0.102±0.006 m).

#### Honeybees use the stepwise modulation of the relative rate of expansion set-point to converge towards a medium set-point

We consequently tested whether these switches towards a new set-point tended to be upwards, downwards or neither. To do so, we determined the change in set-point between all pairs of consecutive constant-*r* segments within a single landing trajectory (Δr*) (Fig. 5). In the 227 landing manoeuvres identified with the constant-*r* segments, 100 landings had more than one constant-*r* segment. Within these 100 landings, we identified 132 pairs of consecutive constant-*r* segments.

Typical examples of these multi-constant-*r* segments are shown in Figs 3A–C and 5B,C. Fig. 3A,B shows cases in which the honeybee upregulated its constant-*r* set-point, whereas Fig. 3C shows landings in which the constant-*r* set-point was downregulated. Fig. 5B,C shows approach flights with two and three identified constant-*r* segments, respectively. For comparison, Fig. 5A shows a landing with a single constant-*r* segment.

The change in set-point (Δr*) between consecutive constant-*r* segments varied over a large range (Fig. 5E), whereby honeybees transitioned 64 times to a lower set-point and 68 times to a higher set-point (examples in Fig. 3A–C). During these transitions, they decreased their set-point with an average step size of Δr*=−0.28±0.32 s^−1^ or increased it on average with a step size of Δr*=0.63±0.62 s^−1^, respectively (means±s.d.).

We used a mixed-effects model to test how these changes in set-point varied with the magnitude of the first constant-*r* segment of each pair (Fig. 5F; Table S3). Independent of visual texture and distance from the platform, the change in set-point varied linearly with the magnitude of the set-point, with a negative slope (dΔr*/dr*=−0.433±0.085, *P*<0.001), and with a zero change in set-point (Δr*=0 s^−1^) at r*=3.14 s^−1^. Thus, honeybees tended to stepwise increase their set-point when flying at a set-point smaller than the *r*_0_*=3.14 s^−1^, and switched to a lower set-point when operating at a set-point larger than *r*_0_*; in addition, the shift magnitude varied linearly with the difference between the current expansion rate set-point and the so-called switch-reversal set-point (*r*_0_*). Note that the slope dΔr*/dr*=−1 would lead to honeybees reaching the switch-reversal set-point (*r*_0_*) in one step. Because the slope dΔr*/dr*=−0.433 is less than−1, a honeybee will on average converge only 43% towards this switch-reversal set-point in one step.

This shows that landing honeybees tend to fly at a constant relative optical expansion rate for a certain period of time. The duration of this period is dependent on the magnitude of the constant-*r* set-point, whereby the duration is longer at medium set-points as compared with the low set-points. When switching from one set-point to the next, they tend to converge on average 43% towards the set-point *r*_0_*=3.14 s^−1^, which lies in the medium r* set-point group (2.58<r*≤3.29 s^−1^).

#### Dependence of relative rate of expansion set-points on distance from the landing platform

In 227 landing manoeuvres identified with the constant-*r* segments, we further tested how the set-point of relative rate of expansion varied with distance to the landing platform (Fig. 5A–D). Similar to our previous bumblebee study (Goyal et al., 2021), we found a linear relationship between the logarithmic transformations of the set-points r* and the mean distance to the surface y* (Fig. 5D and Table 2; Fig. S3). We used a linear mixed-effects model to find an estimate of the slope *m* of this linear variation. The model predicted that honeybees, on an average, increased their set-point with decreasing distance to the surface at a rate *m*=−0.258±0.029 (mean±s.e.m.). This *m* is equivalent to a parameter time-to-contact rate 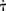 used to describe the landing strategy of birds (Lee et al., 1991; 1993; Whitehead, 2020). This average increase in set-point with reducing distance from the platform can be explained by the fact that the average stepwise increase between consecutive constant-*r* segments was 2.25 times larger than the average stepwise decrease (average stepwise increase: Δr*=0.63±0.62 s^−1^; average stepwise decrease: Δr*=−0.28±0.32 s^−1^).

#### Expansion cues influence the mean set-point at which honeybees fly during landing

**Fig. 6.**
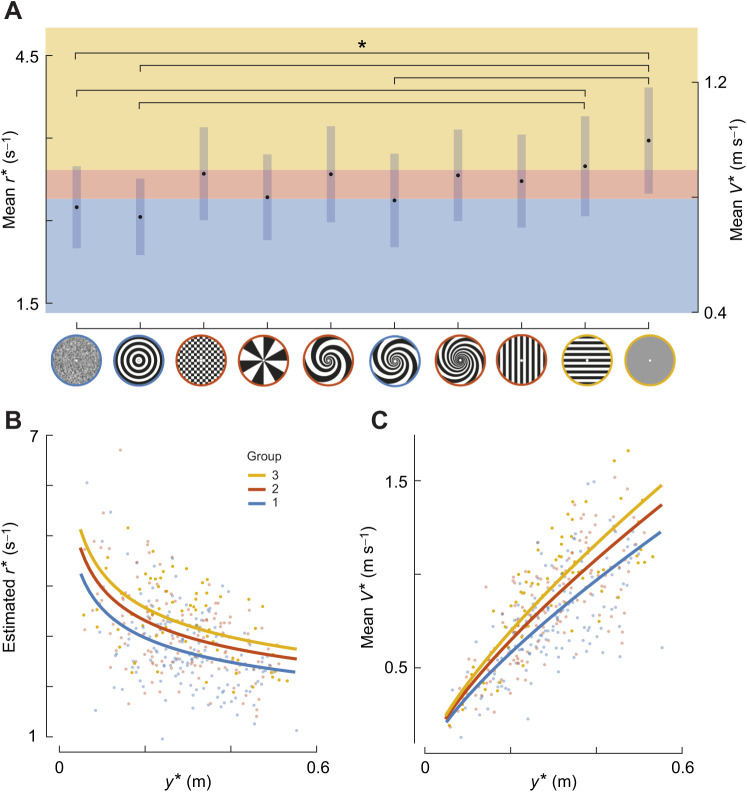
**The mean set-points of relative rate of expansion at which honeybees land varies with the type of landing platform.** (A) The mean set-points of relative rate of expansion r* for honeybees landing on platforms with the 10 different visual patterns. The axis on the right shows the corresponding mean approach velocity V* at the average distance from the landing platform (y*=0.287 m). Black dots and vertical bars indicate the estimated means and 95% confidence intervals, respectively. Significant differences between patterns are indicated by brackets (*P<0.05; Table 2). The patterns are grouped into three clusters, based on the set-point of relative rate of expansion r* that the patterns elicit in the bees. The low-r* cluster (blue) is defined as the group with r* significantly different from the highest r* pattern (grey pattern), and consists of the random Julesz, rings and 4-arm spiral patterns. The high-r* cluster (yellow) is similarly defined as the group with r* significantly different from the lowest r* pattern (ring pattern), and consists of the horizontal stripes and grey pattern. All other patterns fall within the intermediate-r* cluster (orange). We used the same clusters in the average landing analysis (Fig. 2). (B,C) The set-points of optical expansion rate r* (B) and the mean approach velocity V* (C) with mean distance to the landing platform y*. The data points show results for all identified set-points of relative rate of expansion and are colour coded according to the groups defined in A. The number of landings and sampled individuals per treatment are given in Table S1.

The linear mixed-effects model between the set-points r* and the mean distance to the surface y* also allowed us to predict how the observed set-points varied among different landing patterns (Fig. 6). As for the average approach analysis, we found that honeybees exhibited higher set-points, and thus flew faster towards the landing platform (during the constant-*r* segments) when presented with the horizontal stripes (r*=3.16±0.20 s^−1^) and the grey pattern (r*=3.47±0.28 s^−1^). Moreover, they exhibited lower set-points, and thus flew slower (during the constant-*r* segments) in the presence of the random Julesz (r*=2.66±0.18 s^−1^), ring (r*=2.54±0.19 s^−1^) and 4-arm spiral pattern (r*=2.75±0.16 s^−1^). The set-point r* in the presence of other landing patterns fell between these two groups (Fig. 6A,B, Table 2).

Among the tested landing platform patterns, honeybees exhibited 30%, 37% and 26% higher mean set-point r* in the presence of the grey pattern as compared with the random Julesz, ring and 4-arm spiral patterns, respectively. Additionally, honeybees exhibited 19% and 24% higher mean set-point r* in the presence of the horizontal stripes as compared with the random Julesz and ring patterns, respectively (Fig. 6A, Table 2). All comparisons among other landing patterns were statistically insignificant. Note that this increase in the mean set-point results in an equivalent increase in the mean approach velocity V* at the average distance from the landing platform (y*=0.287 m).

## DISCUSSION

Here, we examined how honeybees use optical expansion cues to control their landing on vertical platforms with different levels of visual information. These types of landings are expected to be similar to the landings in nature when honeybees fly between flower patches or when they land on a hive entrance. Honeybees also regularly land on horizontal surfaces. For such landings, optical expansion cues are used to control the vertical descent speed, whereas translatory optic flow cues can be used to reduce the forward flight speed throughout the manoeuvre (Expert and Ruffier, 2015; Franceschini et al., 2007; Srinivasan et al., 2000; Whitehead et al., 2023). Although we analysed landings on vertical surfaces only, optical expansion cues are elicited during an approach towards any surface, ranging from vertical to horizontal, and objects of a large variety of shapes (Baird et al., 2013). Whether the landing control strategy described here for approaching a vertical object is also exhibited for all these other conditions remains to be tested.

To find out how honeybees land on vertical surfaces, we used two complementary methods to analyse 309 recorded landing manoeuvres on surfaces that presented different types of optical expansion cues. These are the analysis of average landing trajectories per treatment as developed by Baird et al. (2013) and the analysis based on the individual landing trajectories as developed by Goyal et al. (2021).

### The average landing approach of honeybees

The average landing trajectory analysis revealed that honeybees reduce their velocity linearly with distance to the surface during their approach towards a landing platform, and thereby keep the relative optical expansion rate approximately constant. This suggests that, on average, honeybees approach a landing surface by flying at a single constant relative rate of optical expansion. These results are in concurrence with those of previous studies on the landing strategies of honeybees (*Apis mellifera ligustica*: Baird et al., 2013) and bumblebees (*Bombus impatiens*: Chang et al., 2016; *Bombus terrestris*: Goyal et al., 2021).

The value of the mean set-point r* predicted in this analysis (r*=3.26±0.16 s^−1^) is similar to that reported previously in honeybees (r*=3.11 s^−1^, computed from fig. 2 in Baird et al., 2013). This is also true for the mean set-point r* in the presence of the landing patterns, for which the data are same in the present study and Baird et al. (2013) (5%, 3% and 10% difference for ring, checkerboard and spoke patterns, respectively).

### The landing strategy of individual honeybees

The average trajectory analysis of landing honeybees in this study provided a useful insight into how they vary their mean approach velocity with distance to the landing surface, but it failed to capture the detailed variations in flight kinematics observed during individual landings (compare Fig. 2A,B with Fig. 5A–C). Specifically, it missed the multiple set-points of relative rate of expansion that honeybees exhibit during their approach and instead suggested that they flew at a single set-point throughout their approach.

Individual honeybees could land using three different landing strategies: either the single constant-*r* or stepwise constant-*r* strategies used by landing insects, or the constant-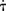 strategy of birds. We tested which one of the three models fitted the landing dynamics of honeybees best by using an AIC selection procedure (Fig. 4). The stepwise constant-*r* strategy resulted in the best fit, despite it being punished by the larger number of fitting parameters. This suggests that, like bumblebees, honeybees land by regularly switching between various constant-*r* set-points.

To capture these multiple set-point dynamics, we applied the individual track-based analysis method we developed (Goyal et al., 2021) to all individual recorded landings. Using this, we identified 227 landing manoeuvres with 359 track segments in which honeybees controlled their deceleration to achieve a constant relative rate of expansion (Fig. 3; see also Database S2 in Dryad, https://doi.org/10.5061/dryad.ghx3ffbsj). Analysing these showed that, on average, honeybees increase their set-point r* with decreasing distance to the surface y*, and that this variation is well captured by a linear fit between their logarithmic transformations with negative slope *m*=−0.258±0.029 (Fig. 5D).

Note that if the honeybees had used a constant-*r* landing strategy as previously suggested (Baird et al., 2013), this analysis approach should have yielded an r* distribution independent of distance to the landing surface (slope *m*≈0). Thus, the significantly negative correlation between set-point r* and y* (Fig. 5D) provides further evidence that the landing honeybees use a stepwise landing strategy similar to that of bumblebees.

When switching to a new optical expansion rate set-point, honeybees need to accelerate or decelerate quickly to converge on the new set-point. Here, we did not study how honeybees achieve this, but our recent study on these transient responses in bumblebees suggests that the flight phase at the optical expansion rate set-point and the preceding transient acceleration and deceleration phases results from the same visual-motor control system (Goyal et al., 2022a). This suggests that also for honeybees, the transient responses prior to flying at a constant optical expansion rate set-point are regulated using the same visual-motor control system.

### The set-point switch behaviour of honeybees

In addition to revealing their landing strategy, the analysis of individual manoeuvres describes how honeybees switch between the set-points of relative rate of expansion. Our result shows that honeybees are more likely to increase their set-points when they fly at a set-point lower than the switch-reversal set-point (*r*_0_*=3.14 s^−1^), and they tended to switch to a lower set-point when operating at a set-point value higher than *r*_0_* (Fig. 5F). Moreover, the set-point switching magnitude (Δr*) depends linearly on the current optical expansion rate set-point (r*) with a slope of dΔr*/dr*=−0.433±0.085. Thus, when switching to a new optical expansion rate set-point, honeybees tended to converge for on average 43% towards the switch-reversal set-point of *r*_0_*=3.14 s^−1^. Because most landing honeybees started their landing approach at r* values lower than *r*_0_*=3.14 s^−1^ (Fig. 5F), the average landing honeybee increased its optical expansion rate set-point with reducing distance from the platform (Fig. 5D).

The dynamics of stepwise convergence towards an average optical expansion rate set-point *r*_0_*=3.14 s^−1^ allows honeybees that start their landing approach at a large range of flight speeds (Fig. 3D) to convert to a relatively narrow band of approach velocities closer to the platform (Fig. 6C). This might be important for making successful, efficient and controlled landings.

### Expansion cues of the landing platform affect the landing strategy of honeybees

We varied the expansion cues that honeybees use during landing by using different visual landing platform patterns. Among the tested patterns, both the average trajectory analysis per treatment and the analysis of individual tracks (Figs 2 and 6, respectively) show that honeybees exhibited the highest set-points, and thus flew fastest, when landing on the grey platform and the horizontal stripe platform (high-r* cluster); they flew slowest when landing on the random Julesz, ring and 4-arm spiral platforms (low-r* cluster). When landing on these last three patterns, honeybees flew on average 30%, 37% and 26% slower than when landing on a grey platform, respectively. This relatively large reduction in flight speed suggests that these differences were not only statistically significant (Table 2) but also biologically relevant.

Because the grey pattern offers weaker expansion cues than the random Julesz, ring and 4-arm spiral platforms, the observed behaviour is in agreement with the results from earlier investigations which show that insects, including honeybees, fly faster when front-to-back translatory optic cues are reduced (Baird, 2005; Baird et al., 2010, 2011, 2020, 2021; Barron and Srinivasan, 2006; Linander et al., 2015). Moreover, honeybees exhibit similar approach velocities in the presence of horizontal stripes and the grey pattern (high-r* cluster). This suggests that honeybees do not parse the vertical expansion flow well, something that may be a limitation of their sensory system, as pure vertical expansion cues would rarely be encountered by honeybees flying in natural conditions.

Our results also show that honeybees use a similar landing approach in the case of the 3-, 4- and 6-arm spiral patterns. This suggests that honeybees can measure the relative rate of expansion largely independently of the spatial frequency content in the landing patterns. This finding is consistent with other flight behaviours of honeybees, where they use angular velocity of the image and are insensitive to the spatial content in the image (Baird, 2005; Baird et al., 2013; Si et al., 2003; Srinivasan, 1992; Srinivasan et al., 1991, 1996).

### Comparison between the modular landing strategies of honeybees and bumblebees

**Fig. 7.**
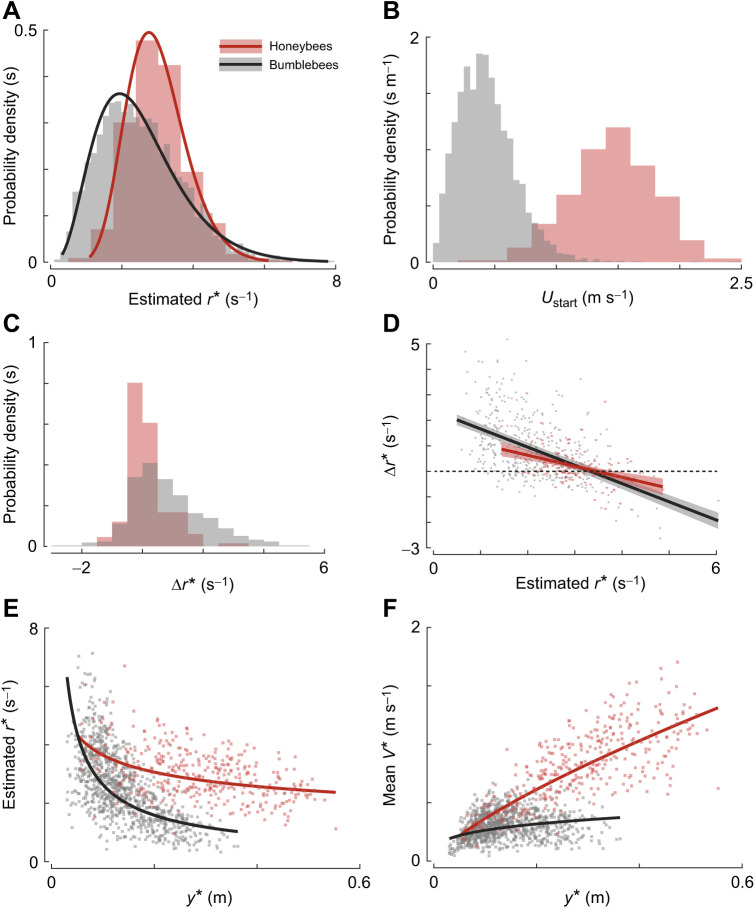
**Comparison of the landing strategies observed in honeybees and bumblebees.** Data for honeybees (red) are from this study; those for bumblebees (grey) are from Goyal et al. (2021). (A–C) The probability density of (A) the set-points of relative rate of expansion r*, (B) the starting 3D flight speeds *U*_start_ and (C) the change in set-point between two consecutive constant-*r* segments Δr*, for the landings of honeybees and bumblebees. (D) The change in set-point Δr* with the set-point magnitude r* for honeybees and bumblebees (Table S3). (E,F) The set-points of relative rate of expansion r* (E) and the mean approach velocity V* (F) with mean distance to the landing platform y* for honeybees and bumblebees as they approached a landing surface. The datapoints in D–F show the results of all identified constant-*r* segments (*n*=359 and *n*=2042 for honeybees and bumblebees, respectively). The curves show the statistical model outputs (and 95% confidence intervals in D). The bumblebee results are for bumblebees landing from free-flight on a vertical landing platform in sunrise light conditions (144.9 lx), whereby results are averaged over checkerboard and spokes patterns (see Goyal et al., 2021, for details). Note that the solid lines in E and F also correspond to the theoretical curves that would result from following the constant time-to-contact rate 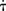 landing strategies suggested in birds (Lee et al., 1991, 1993; Whitehead, 2020).

The modular landing strategy of honeybees described here is also exhibited by bumblebees (*B. terrestris*) (Goyal et al., 2021), with several key similarities and differences (Fig. 7). First, both honeybees and bumblebees exhibit a range of set-points during landing with distributions that can be captured by gamma distributions. However, honeybees exhibit a narrower distribution and a higher mean set-point than bumblebees (Fig. 7A). Second, both increase their set-point with reducing distance to the surface, but honeybees increase it at a higher rate (*m*=−0.26±0.03) than bumblebees (*m*=−0.73±0.01) (Fig. 7E).

Another key aspect for comparison is the set-point switching behaviour of honeybees and bumblebees. For this purpose, we first analysed this behaviour in bumblebees (see Supplementary Materials and Methods, Statistical models) as it was not reported in Goyal et al. (2021). Hereby, we used landing manoeuvres of bumblebees in which they landed from a free-flight condition in the highest tested light condition (data available from Mendeley: doi:10.17632/rrbjyhkm8z.1), as this is most similar to the conditions for the honeybees.

The most striking result is that the average switch-reversal set-point of honeybees and bumblebees is similar (r*=3.1 s^−1^ and r*=3.0 s^−1^, respectively). This shows that both bee species stepwise converge towards an optical expansion rate r*=3 s^−1^ when approaching the landing platform. The stepwise change in the set-point (Δr*) at which bumblebees do this is 63% lower in honeybees than in bumblebees, as indicated by the differences in slopes (dΔr*/dr*) in Fig. 7D. Also, bumblebees switched to a higher set-point more often (73%) as compared with honeybees (52%), and exhibited a wider Δr* distribution (Fig. 7C).

Finally, by using a stepwise constant-*r* landing strategy, bumblebees land 16% faster than if they used a single constant-*r* landing strategy (Goyal et al., 2021). Using the same analysis approach, we found that our honeybees only landed 4% faster using the stepwise constant-*r* landing strategy (approach speed ratio *V*_stepwise_/*V*_single_=1.04±0.12, where *V*_stepwise_ and *V*_single_ are the mean approach flight speed for the stepwise and single constant-*r* landing strategies, respectively). The reduced speed ratio for honeybees compared with bumblebees can be explained by their higher initial flight speed (Fig. 7B), which causes the honeybees to upregulate their expansion rate set-point less often than the bumblebees did (Fig. 7C).

The differences in the initial speeds are probably a combined result of the differences in light intensity and the maximum distance available in front of the landing platforms. To ascertain the exact cause of these differences, an additional study is needed wherein the landing manoeuvres of different insects are recorded with systematic variation in environmental conditions and distance in front of the landing platform.

### Stepwise regulation of set-point of optical expansion rate approximates the constant-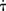 landing strategy of birds

The dependence of set-point of optical expansion rate on distance to the landing surface is captured by a linear relationship between their logarithmic transformations (Fig. S3). The slope *m* of this linear relationship is referred to as a time-to-contact rate 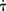 (Baird et al., 2013; Goyal et al., 2021), and is equivalent to the parameter 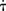 used in the literature to describe the landings of birds (Baird et al., 2013; Lee et al., 1991, 1993; Whitehead, 2020). Therefore, the landing strategies of bees identified in this study can be recognized as a discrete approximation of this landing strategy of birds. This is because the visual guidance strategy of bees results in a stepwise increase of the optical expansion rate with reducing distance to the surface, whereas the visual guidance of birds results in a continuous increase in the optical expansion rate with reducing distance.

Unlike birds, flying insects cannot use binocular stereopsis or focal adjustment to estimate their distance to a landing substrate, at least not during their approach flight (Srinivasan, 1992). Landing insects might use their modular landing strategy to approximate a constant-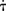 strategy without the need to have a continuous estimate of distance from the surface. This helps landing bees to deal with challenging environmental conditions (e.g. low light), and for slow-flying animals to reach their target faster. Both are beneficial for their foraging efficiency and survival.

The dependence of set-point on distance in the here-described honeybee landing strategy suggests that bees still might need a discrete estimate of distance to the surface while landing, each time the animal switches to a new optical expansion rate set-point. This observation is especially pertinent as optical flow cues, such as visual expansion, provide the ratio of velocity and distance, but not these quantities separately. There are different possibilities on how these insects may estimate distance to the surface without using binocular stereopsis (Baird et al., 2021; Corke and Good, 1992; Croon and De Croon, 2016; de Croon et al., 2021; Ho et al., 2017; van Breugel et al., 2014), but more research is needed to find the underlying mechanism used in this context.

### Conclusion

Honeybees use visual expansion cues to land and reach their food sources. Based on their average approach, it was previously proposed that they perform such landings by holding the relative rate of expansion approximately constant at a single value (referred to as set-point) throughout their landing approach. Here, we performed an additional individual track-based analysis to show that honeybees instead use a range of expansion rate set-points, and regularly adjust their set-point in a stepwise manner. Moreover, we found that honeybees use a set-point switching mechanism that allowed them to converge to stereotypic landing conditions close to the surface, for a large range of initial flight speeds and visual landing platform patterns.

The presence of this modular landing strategy in bumblebees (Goyal et al., 2021) and now also honeybees suggests that it is likely to be found in other flying insects that use visual cues to land. Furthermore, our results can help guide searches for the neural circuits that underlie landing control, and they can inspire similar landing control strategies for robotic flying systems. Finally, this study highlights the importance of using rigorous individual-based analysis methods, instead of average kinematics analyses, when aiming to generate a mechanistic understanding of animal behaviour, biomechanics and sensory-motor control.

## Supplementary Material

10.1242/jexbio.245956_sup1 Supplementary information
