# Supplementary material for: Visual guidance of honeybees approaching a vertical landing surface
Source: J Exp Biol. 2023 Sep 1;226(17):jeb245956. doi: 10.1242/jeb.245956 (PMC10482386; doi:10.1242/jeb.245956)
Supplement: Supplementary information [file jexbio-226-245956-s1.pdf]

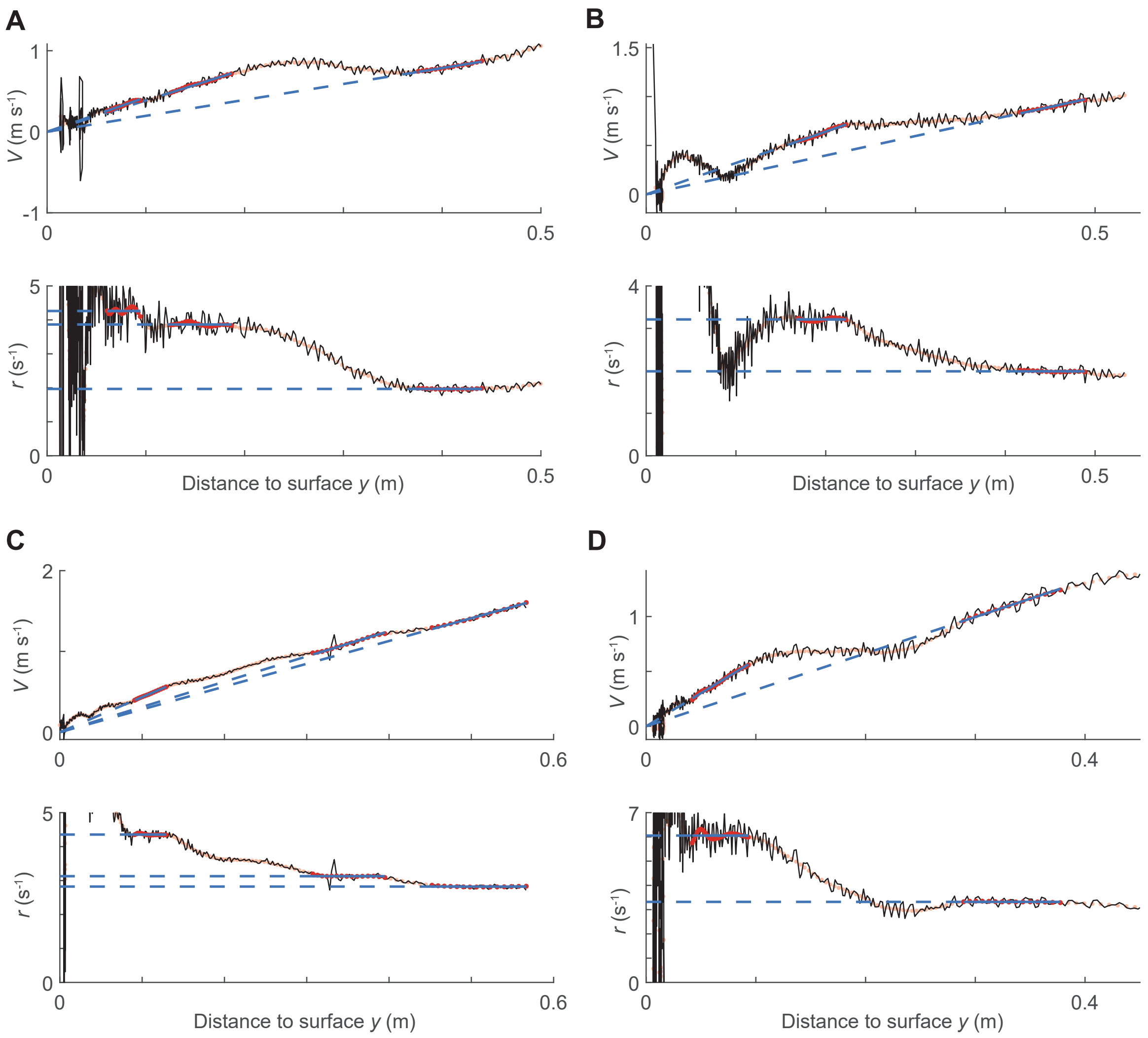

Fig. S1. The comparison of raw data with the low-pass filtered data. Approach velocity V and relative rate of expansion r with perpendicular distance to the landing platform y for a landing approach towards a checkerboard pattern (A), gray pattern (B), horizontally striped pattern (C) and random Julesz pattern (D). In each panel, the identified constant-r segments are highlighted in red and the raw data (black) is overlayed on the low-pass filtered data (orange).

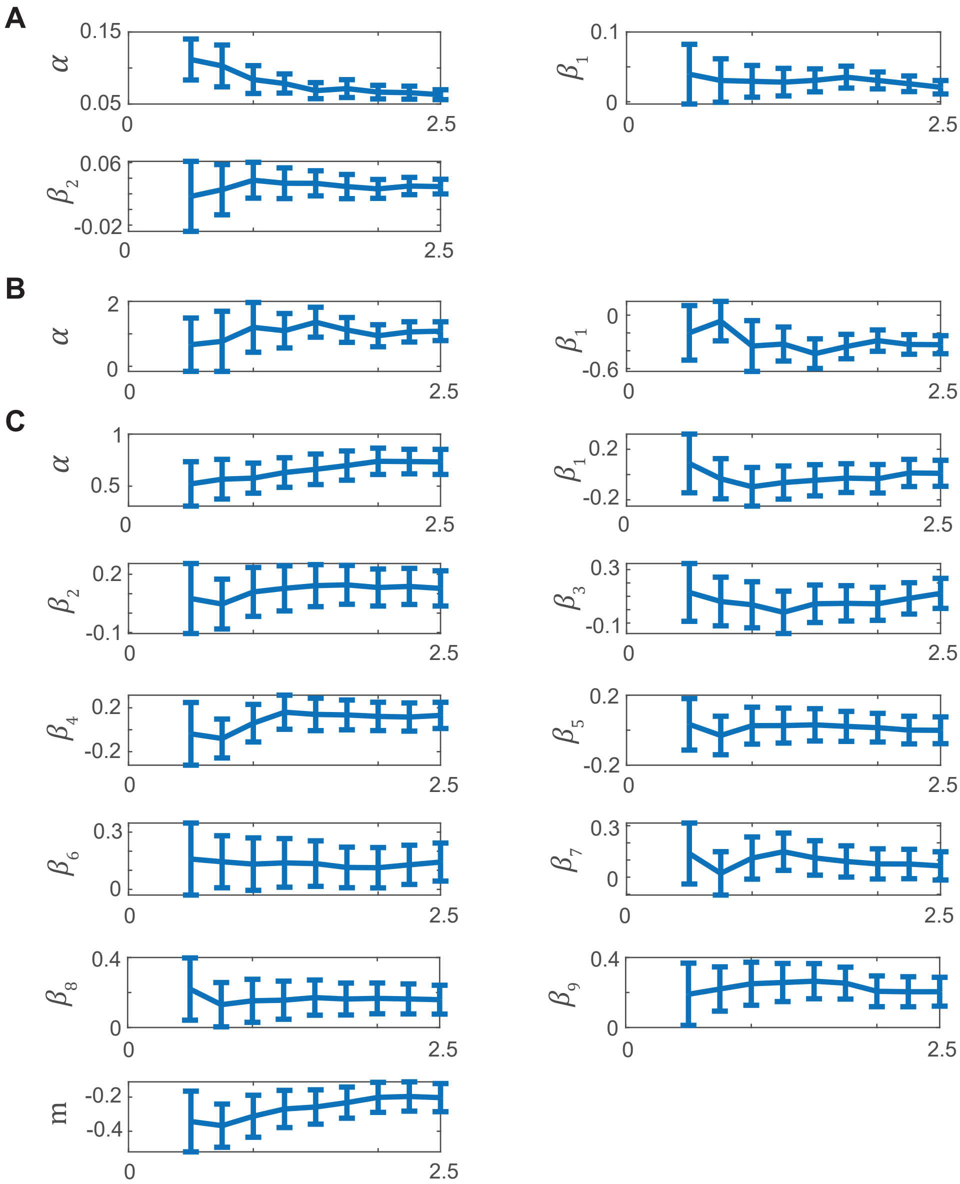

Fig. S2. The effect of factor *f* on the results. (A) The distance covered by honeybees Δy^* with three regions of optical expansion rate set-point r^* for different factors f (Equation S2: $\Delta y^*_{i,s,f} \sim N(\alpha + \alpha_s + \alpha_f + \beta_1\ \mathrm{MEDIUM}_{i,s,f} + \beta_2\ \mathrm{HIGH}_{i,s,f},\ \sigma^2)$). The similar results are observed for the travel time Δt^*. (B) The difference between the new and the current set-point Δr^* with the current set-point r^* for different factors f (Equation S3: $\Delta r^*_{i,s,f} \sim N(\alpha + \alpha_s + \alpha_f + \beta_1\ r^*_{i,s,f},\ \sigma^2)$). (C) The dependence of the set-point r^* on distance to the platform y^* along with the effect of different landing patterns for different factors f (Equation S4: $\log(r^*_{i,s,f}) \sim N(\alpha + \alpha_s + \alpha_f + \sum_{j=1}^{9} \beta_j\ \mathrm{PATTERN}_{j,i,s,f} + m\ \log(y^*_{i,s,f}),\ \sigma^2)$). (A–C) The vertical bars for each coefficient indicate 95% confidence intervals.

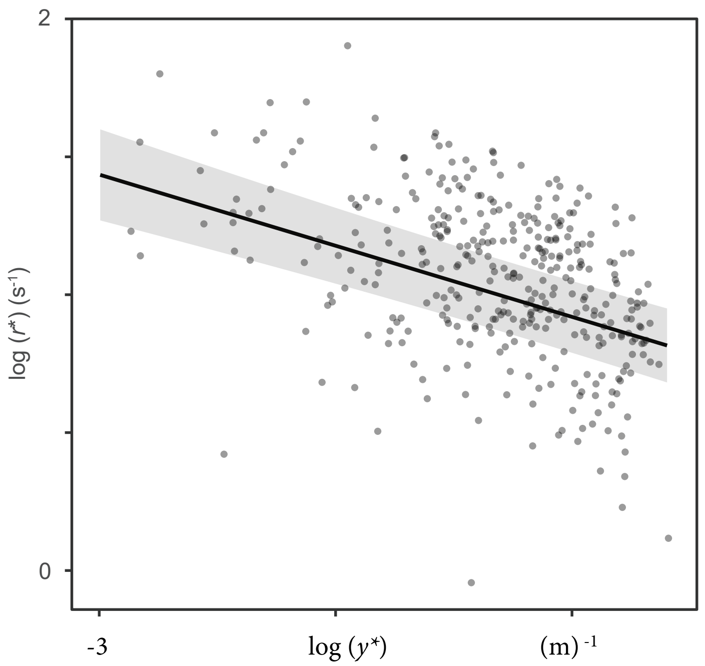

Fig. S3. The set-point of optical expansion rate r^* with distance to the surface y^* in the logarithmic domain as identified by the linear mixed-effects model in Equation S4 (Table 2).

Table S1. The number of landing maneuvers and honeybees recorded in each tested treatment, and the number of landing maneuvers that are identified with constant-*r* segments for different values of factor *f*.

Landing pattern	Number of landings	Number of honeybees	Factor *f*								
			0.5	0.75	1	1.25	1.5	1.75	2	2.25	2.5
Random Julesz	31	15	15	21	23	24	26	28	30	30	30
Ring	21	7	4	8	11	14	14	15	15	17	18
Checkerboard	22	10	8	15	18	19	20	20	21	22	22
Spoke	19	15	6	6	7	7	10	10	13	15	16
3-arm Spiral	19	11	3	7	10	11	11	13	15	15	16
4-arm Spiral	67	12	26	36	46	50	56	56	57	59	61
6-arm Spiral	21	12	7	11	13	16	16	18	18	18	19
Vertical stripes	43	8	11	18	23	28	30	31	32	36	38
Horizontal stripes	44	10	14	25	26	28	31	33	36	37	38
Grey	22	10	4	8	12	12	13	14	16	19	19
Total landings	309		98	155	189	209	227	238	253	268	277

Journal of Experimental Biology • Supplementary information

Table S2. The analysis of distance covered and travel time of honeybees at different set-points of relative rate of expansion. The data is extracted from 359 constant-r segments in 227 landing maneuvers of honeybees (factor $f = 1.5$). The post-hoc tests compare differences between the mean distance covered (Δy^*) or the mean travel time (Δt^*) in three set-point regions: low ($r^* \leq 2.58$ s^{-1}), medium (2.58 s$^{-1} < r^* \leq 3.29$ s^{-1}) and high ($r^* > 3.29$ s^{-1}) (statistical model as given by Equation S2: $\Delta y^*_{i,s,f}$ or $\Delta t^*_{i,s,f} \sim N(\alpha + \alpha_s + \alpha_f + \beta_1 \text{ MEDIUM}_{i,s,f} + \beta_2 \text{ HIGH}_{i,s,f}, \sigma^2)$).

Effect on Δy^*

Fixed effect	Estimate	Std error	t value	Pr(>|t|)
α	0.069	0.006	12.053	$2.73E-28$
β_1	0.031	0.008	3.659	0.000291
β_2	0.033	0.008	4.083	$5.49E-05$
Post-hoc contrasts*	Estimate	Std error	z ratio	p value
Low - Medium	-0.031	0.01	-3.55	$1.32E-03$
Low - High	-0.033	0.01	-4.03	$2.09E-04$
Medium - High	-0.003	0.01	-0.32	1

Effect on Δt^*

Fixed effect	Estimate	Std error	t value	Pr(>|t|)
α	0.100	0.009	11.294	$4.29E-15$
β_1	0.033	0.013	2.589	0.010
β_2	0.021	0.013	1.675	0.095
Post-hoc contrasts*	Estimate	Std error	z ratio	p value
Low - Medium	-0.033	0.013	-2.51	0.037
Low - High	-0.021	0.013	-1.65	0.298
Medium - High	0.012	0.013	0.92	1

Journal of Experimental Biology • Supplementary information

Table S3. The analysis of transition between the set-points of relative rate of expansion observed in honeybees and bumblebees during their approach towards a landing surface.

For honeybees, the data is extracted from 132 transitions in 100 landing maneuvers with more than one constant-r segments (factor $f = 1.5$). (statistical model as given by Equation S3: $\Delta r^*_{i,s,f} \sim N(\alpha + \alpha_s + \alpha_f + \beta_1\ r^*_{i,s,f},\ \sigma^2)$).

Fixed effect	Estimate	Std error	t value	Pr(>|t|)
α	1.36	0.24	5.78	$5.33E-08$
β_1	-0.43	0.08	-5.09	$1.22E-06$

For bumblebees, the data is extracted from 1,122 transitions in 902 landing maneuvers. In these maneuvers, bumblebees landed from a free-flight condition and exhibited more than one constant-r segment. (statistical model as given by Equation S5: $\Delta r^*_{i,d,a,s} \sim N(\alpha + \alpha_d + \alpha_a + \alpha_s + \beta_1\ r^*_{i,d,a,s} + \beta_2\ \text{MEDIUMlight} + \beta_3\ \text{HIGHlight},\ \sigma^2)$).

Fixed effect	Estimate	Std error	t value	Pr(>|t|)
α	1.42	0.11	13.21	$9.57E-05$
β_1	-0.71	0.03	-20.48	$2.56E-79$
β_2	0.32	0.07	4.51	$7.24E-06$
β_3	0.73	0.07	10.24	$1.48E-23$

Journal of Experimental Biology • Supplementary information

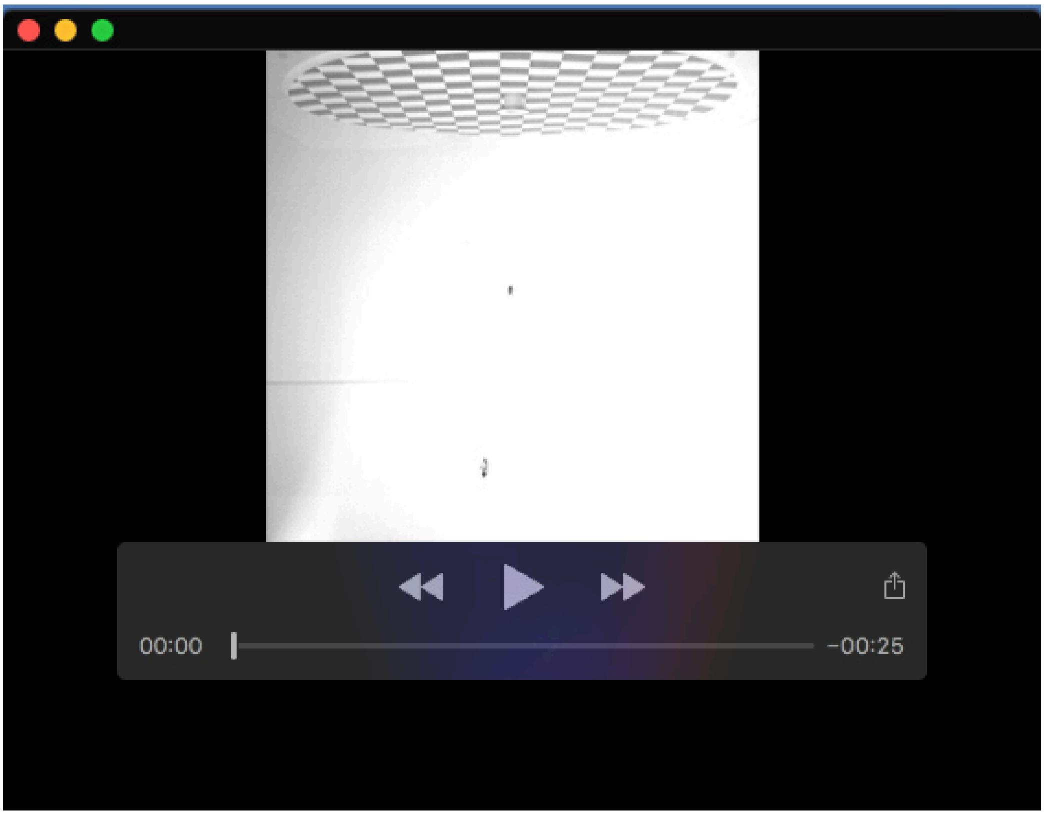

Movie 1. A honeybee landing on a checkerboard pattern, recorded using the top camera. Playback is slowed down 16 times.

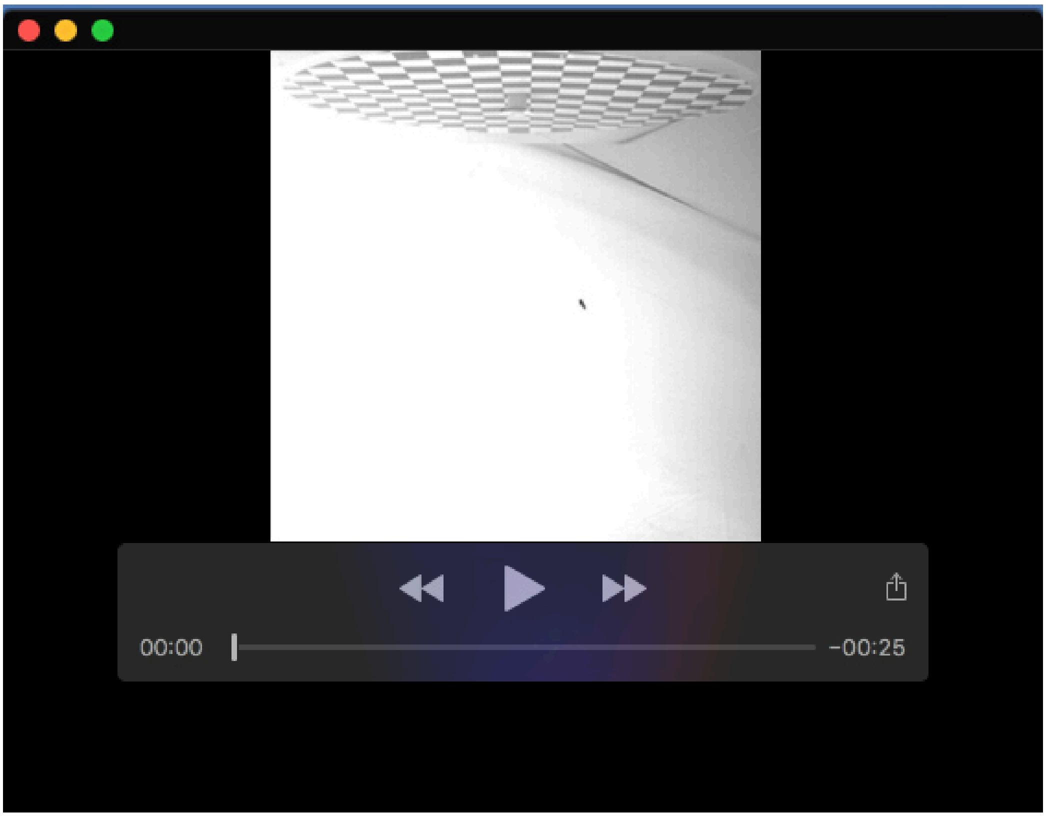

Movie 2. A honeybee landing on a checkerboard pattern, recorded using the side camera. Playback is slowed down 16 times.

Supplementary Materials and Methods

Extraction of set-points of relative rate of expansion

We used an algorithm developed in Goyal et al. [2021a] to extract the constant-r track segments in which honeybees decelerated at an approximately constant relative rate of expansion. For a constant-r segment, three linear regressions are computed - one in the full segment and other two in its equal halves. These linear regressions adjudge the constancy of r with distance to the surface y. The first step of set-point extraction algorithm involves the identification of expected t-distributions of six linear regression parameters (two from each regression). For this purpose, we manually selected 282 constant-r segments in 193 landing maneuvers of honeybees and computed the desired t-distributions. Similar to the bumblebee study, these t-distributions have location parameter μ close to zero and scale parameter σ, which dictates their spread around μ, defined in terms of two parameters: $\sigma_1 = 0.29$ and $\sigma_2 = 1.02$ (see Goyal et al. [2021a] for details). In the second step of set-point extraction algorithm, we used these identified t-distributions to automatically select constant-r segments in the complete dataset. This step computes these six regression parameters in all possible track segments of different sizes (with $15 - 200$ data points) in a landing maneuver and selects those track segments as probable constant-r segments in which these regression parameters lie within a number of scale parameters around their mean values (specified by a threshold factor f). Among these probable segments, a set of non-overlapping segments are identified as constant-r segments. This selection of non-overlapping segments is identified by the root mean square error of the estimated set-point from the observed variation in r. To make this selection similar to the bumblebee study, we reduced the sampling rate of the landing maneuvers of honeybees to 175 Hz.

Statistical models

We developed linear mixed-effects models for both the average-per-treatment and per-track analyses.

The average-per-treatment model

To determine how the mean of relative rate of expansion varied with patterns on the landing disc, we used the following model:

$$r_{i,s,f} \sim N(\,\alpha + \alpha_s + \alpha_f + \textstyle\sum_{j=1}^{9} \beta_j \,\mathrm{PATTERN}_{j,i,s,f},\ \sigma^2) \qquad \text{(S1)}$$

where $r_{i,s,f}$ is the i-th measurement of relative rate of expansion from the f-th flight number in the s-th set, α is the regression intercept for random Julesz pattern (overall intercept), α_s is the set-specific intercept, α_f is the flight-number-specific intercept, $\mathrm{PATTERN}_j$ indicates j-th pattern in the set {Ring, Checkerboard, Spoke, 3-arm spiral, 4-arm spiral, 6-arm spiral, vertical stripes, horizontal stripes, grey}, $\mathrm{PATTERN}_{j,i,s,f}$ indicates if j-th landing pattern is present for the i-th measurement from the f-th flight number in the s-th set (0 = no, 1 = yes), $\beta_j\ \forall\ j \in \{1, 2, \ldots, 9\}$ represent the differences of fixed-effects from the overall intercept, and σ is the residual standard deviation. The statistical output and the results from post-hoc tests for 309 landing maneuvers of honeybees are given in Table 1.

The per-track models

We used four statistical models to report results from the analysis of individual landing maneuvers of honeybees.

1. To find if honeybees preferred to fly at certain set-points of relative rate of expansion, we tested how the distance covered (Δy^*) or travel time (Δt^*) during each set-point varied with the three set-point regions: low ($r^* \leq 2.58\ \mathrm{s}^{-1}$), medium ($2.58\ \mathrm{s}^{-1} < r^* \leq 3.29\ \mathrm{s}^{-1}$) and high ($r^* > 3.29\ \mathrm{s}^{-1}$) along with the landing patterns. The model dredging revealed that the landing patterns did not influence this

Journal of Experimental Biology • Supplementary information

relationship. Therefore, we used the following reduced model:

$$\Delta y^*_{i,s,f} \text{ or } \Delta t^*_{i,s,f} \sim N(\,\alpha + \alpha_s + \alpha_f + \beta_1\,\text{MEDIUM}_{i,s,f} + \beta_2\,\text{HIGH}_{i,s,f},\ \sigma^2) \tag{S2}$$

where $\Delta y^*_{i,s,f}$ or $\Delta t^*_{i,s,f}$ is the i-th measurement from the f-th flight number in the s-th set, α is the regression intercept for low set-point region (overall intercept), α_s is the set-specific intercept, α_f is the flight-number-specific intercept, $\text{MEDIUM}_{i,s,f}$ and $\text{HIGH}_{i,s,f}$ indicate if that measurement belongs to the medium and high set-point region, respectively (0 = no, 1 = yes), $\beta_j\ \forall\ j\ \in\ \{1, 2\}$ represent the differences of fixed-effects from the overall intercept, and σ is the residual standard deviation. The statistical output and the results from post-hoc tests are given in Table S2.

2. To find how honeybees selected the new set-point of relative rate of expansion, we analyzed the transition between two consecutive set-points in landing maneuvers with more than one constant-r segments. Specifically, for each transition, we tested how difference between the new and the current set-point (Δr^*) varied with the current set-point (r^*), the distance at which the current set-point is observed (y^*), and the landing patterns. The model dredging revealed that y^* and landing patterns did not influence the relationship between Δr^* and r^*. Therefore, we used the following model:

$$\Delta r^*_{i,s,f} \sim N(\,\alpha + \alpha_s + \alpha_f + \beta_1\, r^*_{i,s,f},\ \sigma^2) \tag{S3}$$

where $r^*_{i,s,f}$ and $\Delta r^*_{i,s,f}$ are the i-th set-point and the step-change in it, respectively, from the f-th flight number in the s-th set, α and β_1 are the regression intercept and slope, respectively, α_s is the set-specific intercept, α_f is the flight-number-specific intercept, and σ is the residual standard deviation. The statistical output is given in Table S3.

3. To find how honeybees adjusted their set-point (r^*) with distance to the platform (y^*) and landing patterns, we used the following model:

$$\log(r^*_{i,s,f}) \sim N(\,\alpha + \alpha_s + \alpha_f + \textstyle\sum_{j=1}^{9} \beta_j\ \text{PATTERN}_{j,i,s,f} + m\ \log(y^*_{i,s,f}),\ \sigma^2) \tag{S4}$$

where $r^*_{i,s,f}$ and $y^*_{i,s,f}$ are the i-th measurements of the set-point of relative rate of expansion and the mean distance, respectively, from the f-th flight number in the s-th set, α is the regression intercept for random Julesz pattern (overall intercept), α_s is the set-specific intercept, α_f is the flight-number-specific intercept, PATTERN_j indicates j-th pattern in the set {Ring, Checkerboard, Spoke, 3-arm spiral, 4-arm spiral, 6-arm spiral, vertical stripes, horizontal stripes, grey}, $\text{PATTERN}_{j,i,s,f}$ indicates if j-th landing pattern is present for the i-th measurement from the f-th flight number in the s-th set (0 = no, 1 = yes), $\beta_j\ \forall\ j\ \in\ \{1, 2, \ldots, 9\}$ represent the differences of fixed-effects (landing patterns) from the overall intercept, m represents the regression slope for predictor $\log(y^*)$, and σ is the residual standard deviation. The statistical output and the results from post-hoc tests are given in Table 2.

4. To compare how the mechanism of change in a set-point of relative rate of expansion differed between honeybees and bumblebees, we also analyzed the transition between two consecutive set-points in landing maneuvers of bumblebees. For this purpose, we used landing maneuvers of bumblebees in which they landed from a free-flight in the presence of two different landing patterns (spoke and checkerboard) and three different light intensities (low - 13.7 lx, medium - 33.3 lx, high - 144.9 lx) [Goyal et al., 2021b]. First, we identified landing maneuvers that contained set-point transitions [Goyal et al., 2021a]. Then, for these transitions, we tested how the difference between new and current set-point (Δr^*) varied with the current set-point (r^*), the distance at which the current set-point is observed (y^*), the landing patterns and the light conditions. The model dredging revealed that y^* and landing patterns did not influence the relationship between Δr^* and r^*. Therefore, we used the following reduced model:

$$\Delta r^*_{i,d,a,s} \sim N(\,\alpha + \alpha_d + \alpha_a + \alpha_s + \beta_1\, r^*_{i,d,a,s} + \beta_2\,\text{MEDIUMlight} + \beta_3\,\text{HIGHlight},\ \sigma^2) \tag{S5}$$

Journal of Experimental Biology • Supplementary information

where $r^*_{i,d,a,s}$ and $\Delta r^*_{i,d,a,s}$ are the i-th set-point and the step-change in it, respectively, for d-th day ($d \in \{1, 2, \ldots, 14\}$), a-th landing approach ($a \in \{1, 2, \ldots, 902\}$) and s-th landing side ($s = 1$ for hive side and $s = 2$ for food-source side), α is the regression intercept for low light condition (overall intercept), α_d is the day-specific intercept, α_a is the landing-approach-specific intercept, α_s is the landing-side-specific intercept, $\text{MEDIUMlight}_{i,d,a,s}$ and $\text{HIGHlight}_{i,d,a,s}$ indicate if medium light condition and high light condition are present for the i-th measurement from d-th day, a-th landing approach and s-th landing side (0 = no, 1 = yes), β_1 represents the regression slope for predictor r^*, β_2 and β_3 represent differences of fixed-effects from the overall intercept, and σ is the residual standard deviation. The statistical output is given in Table S3.

Bibliography

Pulkit Goyal, Antoine Cribellier, Guido C H E de Croon, Martin J Lankheet, Johan L van Leeuwen, Remco P M Pieters, and Florian T Muijres. Bumblebees land rapidly and robustly using a sophisticated modular flight control strategy. *iScience*, 24(5):102407, 2021a. doi: https://doi.org/10.1016/j.isci.2021.102407.

Pulkit Goyal, Antoine Cribellier, Guido C H E de Croon, Martin J Lankheet, Johan L van Leeuwen, Remco P M Pieters, and Florian T Muijres. Landing manoeuvres of bumblebees. *Mendeley Data*, Version 1, 2021b. doi: https://dx.doi.org/10.17632/rrbjyhkm8z.1.

Journal of Experimental Biology • Supplementary information
